# Exploring the mechanism of *Radix Rhei Et Rhizome* intervention in intracerebral hemorrhage based on systematic pharmacology and proteomics strategy

**DOI:** 10.1042/BSR20201910

**Published:** 2021-03-10

**Authors:** Xiaofei Zhu, Zhiyong Long, Tingting Bao, Liang Liu, Kailin Yang

**Affiliations:** 1 Institute of Integrative Medicine, Department of Integrated Traditional Chinese and Western Medicine, Xiangya Hospital, Central South University, Changsha 410008, China; 2 Shantou University Medical College, Shantou, Guangdong Province, China; 3 Department of Physical Medicine and Rehabilitation, Guangdong General Hospital, Shantou University Medical College, Shantou, Guangdong, China; 4 Xiyuan Hospital, China Academy of Chinese Medical Sciences, Beijing, China; 5 School of Clinical Medicine (Xiyuan Hospital), Beijing University of Chinese Medicine, Beijing, China; 6 People’s Hospital of Ningxiang City, Ningxiang 410600, Hunan Province, China; 7 Graduate College, Capital Medical University, Beijing, China

**Keywords:** Chinese Medicine, Intracerebral Hemorrhage, Radix Rhei Et Rhizome, Systematic pharmacology

## Abstract

**Objective:** To explore the mechanism of *Radix Rhei Et Rhizome* (Dahuang, DH) intervention in intracerebral hemorrhage (ICH) based on systematic pharmacology and proteomics strategy.

**Methods:** The systematic pharmacological strategies were utilized to find the bioactive compounds of *Radix Rhei Et Rhizome*, predict its potential targets, and collect ICH’s disease genes; then, the Cytoscape 3.7.1 software was applied for network construction and network topology analysis. After that, in-depth analysis of the proteomics data of *Radix Rhei Et Rhizome* intervention in ICH was performed to complement and validate the results of systematic pharmacological predictions.

**Results:** A total of three major networks were constructed in the present study: (1) compound–compound target network of *Radix Rhei Et Rhizome*, (2) DH-ICH PPI network, (3) proteomics proteins’ PPI network. These three major networks have been analyzed by network topology, and several small networks derived (such as signaling pathway networks). The enrichment analysis showed that *Radix Rhei Et Rhizome* can intervene in several biological process (such as inflammation, smooth muscle proliferation, platelet activation, blood pressure regulation, angiogenesis, hypoxia, and inflammatory response of leukocytes), signaling pathway (such as FoxO signaling pathway, complement and coagulation cascades, cGMP-PKG signaling pathway, and Rap1 signaling pathway), and reactome pathway (such as signaling by interleukins, interleukin-4 and interleukin-13 signaling, nuclear receptor transcription pathway, and platelet activation).

**Conclusion:** *Radix Rhei Et Rhizome* may intervene in ICH-related biological process, signaling pathway, and reactome pathway found in this research so as to achieve the effect of treating ICH related injuries.

## Introduction

Intracerebral hemorrhage (ICH) refers to bleeding result from non-traumatic rupture of blood vessels in the brain parenchyma, accounting for 25–30% of all strokes. However, its acute mortality rate is 30–40%, and the survivor’s invalidism rate is as high as 70%, which can leave severe complications such as paralysis, aphasia, epilepsy, and dementia, affecting the quality of life of patients [1,2]. The cause of ICH is mainly related to cerebrovascular diseases, such as hypertension with arteriolar sclerosis, microaneurysms or microhemangiomas, and cerebral vascular malformations. Its clinical manifestations vary depending on the location and amount of bleeding; for example, basal nucleus, thalamus, and internal capsule bleeding may cause hemiplegia [2–4]. The ICH-related brain injury can be divided into two categories (ICH primary brain injury and ICH secondary brain injury) due to different mechanisms. ICH primary brain injury is brain parenchymal injury mediated by the hematoma mechanical occupying effect [3,4]. The secondary damage of ICH is mainly due to the strong cytotoxicity of metabolites (including thrombin, hemoglobin, heme, and iron overload, and so on) during the dissolution of hematomas on adjacent brain cells, which triggers inflammatory reactions and oxidative stress, and then causes brain edema and damage to the blood–brain barrier, eventually leading to cell damage and death [4,5]. The current treatment of ICH is mainly surgery. However, surgery can only relieve the mechanical compression of intracranial hematomas, and has no obvious improvement effect on secondary injuries. At present, there is no effective treatment method for secondary injuries. Secondary injuries occur within minutes after the onset of ICH, and can last for several days or even months, which is an important factor leading to cell death in the brain and long-term neurological damage [6].

*Radix Rhei Et Rhizome* (Dahuang, DH) is the dried roots and rhizomes of *Rhympalmatum L, Maximum.exBalf* or *Rhumofificinal Bail* [7]. It is often used in difficult and severe cases in the records of ancient Chinese medicine. The *Yixue Zhongzhong Canxi Lu* by Zhang Xichun clearly records that *Radix Rhei Et Rhizome* can be used to treat ICH. Current systematic reviews and meta-analyses have shown that *Radix Rhei Et Rhizome* can reduce the amount of hematoma, and reduce mortality and severe disability rates [8,9]. Current research has also shown that *Radix Rhei Et Rhizome* and its active compounds have protective effects on the brain nerves, and its mechanism may be related to reducing cerebral hematoma, protecting the blood–brain barrier, inhibiting inflammatory reactions and oxidative stress, and inhibiting apoptosis [10–14]. However, its specific mechanism remains unclear. The development of proteomics and systematic pharmacology has given the opportunity to analyze the molecular mechanisms of *Radix Rhei Et Rhizome* for ICH at the system level [15–17]. At present, researchers do not know much about the modern pharmacological mechanism of Chinese herbal medicine. In order to comprehensively analyze the composition, target, and mechanism of traditional Chinese medicine (TCM) and accelerate the modernization of TCM, systematic pharmacology, the new methodological strategy was developed [15–17]. In order to further explore the substance basis of herbs and herbal formulae interventions in diseases, and to systematically and comprehensively clarify their biomolecular basis for treating diseases, we have developed an integrated strategy based on high-throughput omics and cheminformatics [17–19]. High-throughput omics provides changes in biomolecular networks in animal disease models. Cheminformatics has the advantage of simulating human targets and overcomes the deficiencies of animal experiments. The combination of the two has made breakthroughs in methodology, and also promoted the development of the discipline, providing a good reference for future researchers [19]. Therefore, the present study will integrate proteomics and systematic pharmacology to reveal the therapeutic mechanism of *Radix Rhei Et Rhizome* for ICH and provide reference information for new drugs related to *Radix Rhei Et Rhizome* and its active compounds [17–19].

## Materials and methods

### Potential compounds of *Radix Rhei Et Rhizome*

With “*Radix Rhei Et Rhizome*” as the key word, it was entered into the TCM-related database for potential compound prediction, and several TCM-related databases were applied: TCMSP database (http://lsp.nwu.edu.cn/) [20] and TCM@Taiwan (http://tcm.cmu.edu.tw/zh-tw/) [21]. After searching, a total of 92 *Radix Rhei Et Rhizome* compounds were obtained. Pharmacokinetic parameters [drug-likeness (DL) ≥0.18, Caco-2 permeability> −0.4, and oral bioavailability (OB) ≥30%] were used to screen biologically active compounds, and a total of nine active compounds were obtained [15–19,22–25]. However, since the application of biological models to predict RMP compounds has limitations [26], in order to avoid missing active compounds during the pre-screening process, we searched a large number of references and selected oral absorbable compounds with pharmacological activity.

Eventually, combined with reference [27,28], totally 14 potential targets were obtained: physcion, eupatin, β-sitosterol, daucosterol, mutatochrome, palmidin A, toralactone, emodin, sennoside A, aloe-emodin, (-)-catechin, chrysophanol, and danthron, rhein. SciFinder (http://scifinder.cas.org) and PubChem (https://pubchem.ncbi.nlm.nih.gov/) were used to search the standard molecular structure of those potential targets, and they were drawn in ChemBioDraw 14 and saved in “mol2” format.

### Potential targets of *Radix Rhei Et Rhizome* and ICH genes

The “mol2” format files of the potential compounds were input into PharmMapper server platform (http://lilab-ecust.cn/pharmmapper/) for potential targets prediction [29]. Set “select target set” to “human protein targets only (v2010, 2241)”, and the remaining parameters are default values. The reverse docking prediction results of each compound are downloaded, and the *Z* scores of the docking scores were arranged in descending order. The top 300 targets of each compound were selected for subsequent research. The UniProtKB (http://www.uniprot.org/), a database contains the accurate annotation of proteins and so on, was used for the correction of protein’s names and the collection of official symbols with the species limited to (for potential targets) (Supplementary Table S1) or “Rattus norvegicus” (for proteomics data) (Supplementary Table S2). The ICH genes were collected from GeneCards database (http://www.genecards.org/) [30] and the OMIM database (http://www.ncbi.nlm.nih.gov/omim) [31]. A total of 689 ICH-related genes were obtained. The genes with relevance score> 3 were selected for sequence research (Supplementary Table S3).

The potential targets of *Radix Rhei Et Rhizome* and CI genes were imported into String 11.0 (https://string-db.org/), and the species was restricted to “Homo sapiens” (for potential targets) or “Rattus norvegicus” (for proteomics data) to obtain protein–protein interaction (PPI) data [32].

### Network construction and analysis methods

The active potential compounds, potential targets, ICH genes, and proteomics data were introduced into Cytoscape 3.7.1 (http://www.cytoscape.org/) [33] software to build compound–compound target network of *Radix Rhei Et Rhizome*, DH-ICH PPI network, proteomics proteins’ PPI network, and other small networks derived from these major networks. In the network, nodes represent genes, proteins, or molecules; the connections between nodes are represented by edges, which stands for the interactions among these biological molecules [33]. Degree indicates the number of connections between nodes, while betweenness represents the number of shortest paths through a node [33]. In the DH-ICH PPI network and proteomics proteins’ PPI network, the closely connected parts of the nodes are considered to be the functional area where *Radix Rhei Et Rhizome* plays the role of regulating the biological network, namely Clusters [33]. The Cytoscape’s plug-in MCODE is used for cluster analysis of the network [15–19,33].

### Enrichment analysis methods

The Gene Ontology (GO) enrichment analysis and KEGG signaling pathway enrichment analysis were performed by the Database for Annotation, Visualization and Integrated Discovery (DAVID) v6.8 (https://david-d.ncifcrf.gov) [34]. The reactome pathway enrichment analysis were performed by Reactome Pathway Database (https://reactome.org/) [35]. The biological processes, signaling pathways, and reactome pathways with *P* value <0.05 were collected for analysis.

## Results and discussion

### Potential targets of *Radix Rhei Et Rhizome* and ICH genes

**Figure 1:**
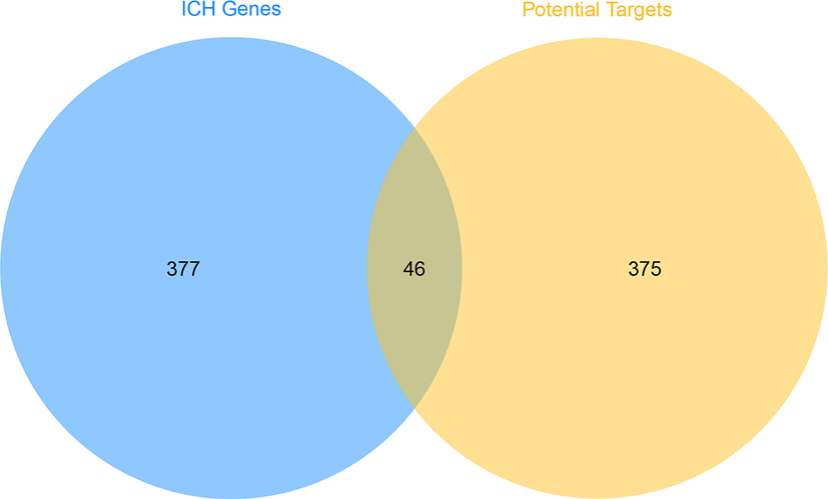
Venn diagram

After introducing 14 potential compounds into pharmmapper for prediction, 425 potential targets were obtained. (-)-Catechin gets 295 potential targets; Aloe-emodin gets 294 potential targets; beta-sitosterol has 217 potential targets; Chrysophanol has 249 potential targets; Danthron has 172 potential targets; Daucosterol has 216 potential targets; Emodin has 293 potential targets; Eupatin has 293 potential targets; Mutatochrome has 251 potential targets; Palmidin A gets 297 potential targets; Physcion gets 294 potential targets; Rhein gets 296 potential targets; Sennoside A gets 297 potential targets; Toralactone gets 296 potential targets. Meanwhile, 423 ICH-related genes with relevance score> 3 were selected for research. There is overlap between the potential target set and the ICH gene set (Figure 1).

**Figure 2:**
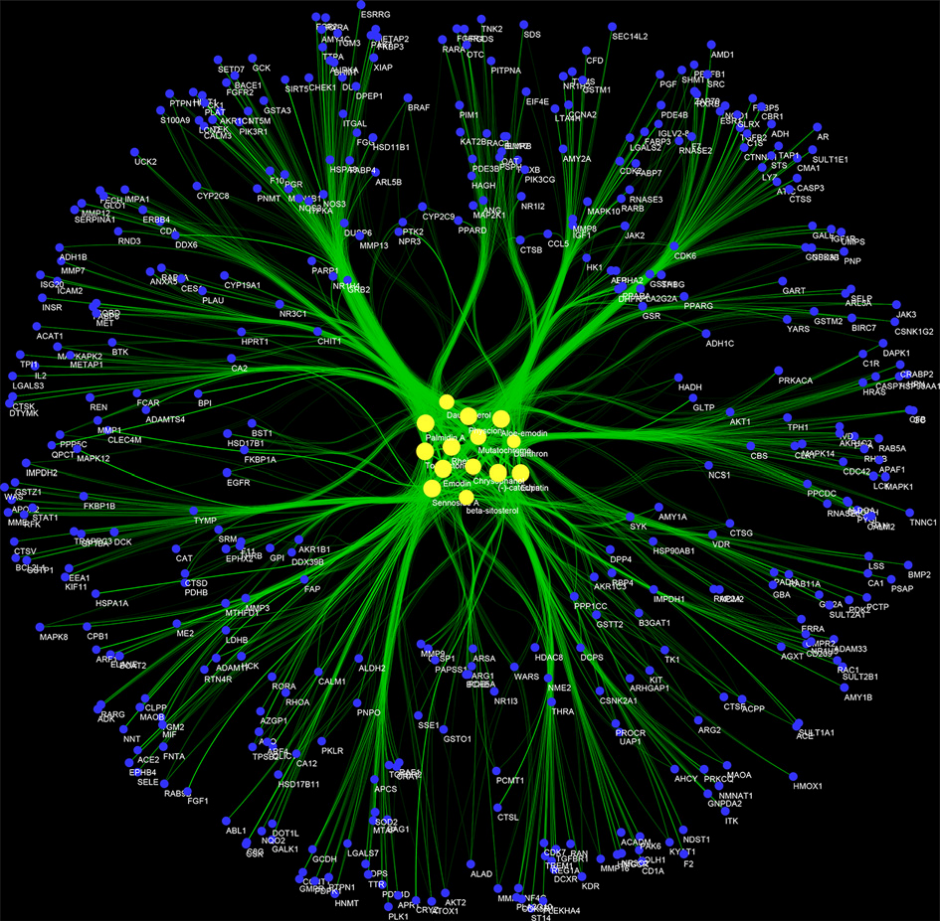
Compound–compound target network of *Radix Rhei Et Rhizome* Yellow and blue circles stand for potential compounds and potential targets, respectively. The larger the node size, the higher the degree of the node. The thicker the line, the greater the edge betweenness of the node.

The potential targets and potential compounds of *Radix Rhei Et Rhizome* were input into Cytoscape 3.7.1 to construct compound–compound target network of *Radix Rhei Et Rhizome*. This network consists of 14 compound nodes, 425 compound target nodes, and 3760 edges. In this network, some targets can be regulated by most compounds, such as: ZAP70, TYMS, TTR, TGM3, SYK, SULT2A1, SRC, RXRA, REN, PTPN1, PRKACA, PLA2G2A, PIM1, PDPK1, PDE5A, PDE4D, PDE4B, NR3C1, NR1H4, NQO1, NOS3; other targets can only be regulated by a small number of compounds (or even only one compound), such as SIRT5, RTN4R, QPCT, PLAT, NPR3, NNT, MAPK12, IGLV2-8, HSPA1A, GLTP, GLRX, FGF1, FCAR, DDX6, CTSL, CRYZ (Figure 2).

### DH-ICH PPI network analysis

#### DH-ICH PPI network construction

**Figure 3:**
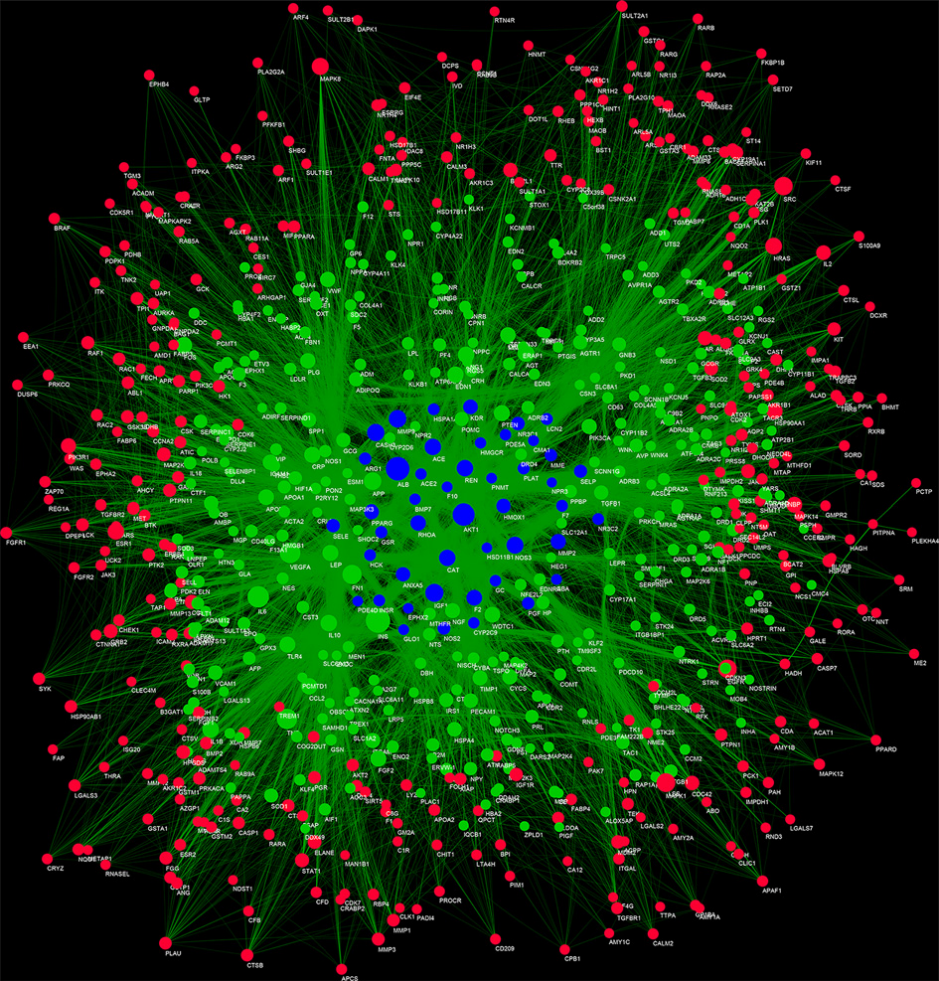
DH-ICH PPI network Blue, green, and red circles stand for DH-ICH targets, ICH genes, and DH targets, respectively. The larger the node size, the higher the degree of the node. The thicker the line, the greater the edge betweenness of the node.

Four hundred and twenty-three (423) ICH genes, 524 potential targets of *Radix Rhei Et Rhizome* (DH), and their PPI data were input into Cytoscape 3.7.1 to establish the DH-ICH PPI network. This network is composed of 331 ICH gene nodes, 370 potential target nodes, 46 DH-ICH target nodes, and 15458 edges. The top 20 targets for degree in this network are: (1) DH targets: MAPK1 (206 edges), SRC (197 edges), EGFR (197 edges), MAPK8 (167 edges), HRAS (158 edges), ESR1 (156 edges); (2) ICH genes: INS (332 edges), IL6 (265 edges), VEGFA (245 edges), TNF (239 edges), FN1 (214 edges), APP (176 edges), AGT (161 edges); (3) DH-ICH targets: ALB (316 edges), AKT1 (279 edges), IGF1 (191 edges), MMP9 (178 edges), CASP3 (174 edges), NOS3 (166 edges), ACE (154 edges) (Figure 3).

#### Biological processes of DH-ICH PPI network

**Figure 4:**
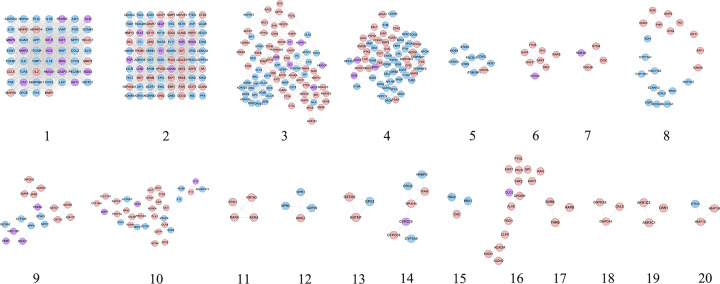
Clusters of DH-ICH PPI network Blue, pink, and purple circles stand for ICH genes, DH targets, and DH-ICH targets, respectively.

The DH-ICH PPI network was analyzed by the MCODE, and 20 clusters were returned (Table 1 and Figure 4). The genes and targets in the top 10 clusters were input into DAVID for GO enrichment analysis, and a lot of biological processes were obtained.

**Table 1:** Clusters of DH-ICH PPI network

Cluster	Score	Nodes	Edges	Targets and genes
1	44.692	53	1162	CCL2, WDTC1, PECAM1, AGT, REN, VCAM1, CASP3, FN1, ICAM1, ADIPOQ, MMP1, PLG, CCL5, IL2, FGF2, CRP, ELN, MAPK8, IL18, SPP1, CAT, MMP2, FOS, MAPK1, LEP, ITGAM, SELE, CYCS, MMP9, TIMP1, PPARG, ALB, VWF, AKT1, HSPA4, APP, IL10, IL6, IGF1, MMP3, TGFB1, MAPK14, TNF, SERPINE1, APOE, EDN1, BCL2L1, IL1B, ACE, HMOX1, NGF, TLR4, NOS3
2	25.873	80	1022	EDN3, EDN2, GRB2, EGFR, PGF, FLT1, GCGR, PGR, ANXA5, RAF1, ADRA1A, ADRA1B, SRC, PLAU, AKT2, AVP, GNB3, AVPR1A, ADRA1D, ELANE, OXT, TAC1, CASP1, KIT, MMP13, CREB1, UTS2, JAK2, STAT1, SOD2, CD40LG, RETN, MMP7, ADAM17, LCN2, ESR1, ITGB1, SOD1, BDKRB2, KNG1, MDM2, MET, PF4, PARP1, AR, AIF1, HSP90AA1, CCNA2, HPGDS, MAP2K1, ENG, LGALS3, TAC3, TACR3, CDC42, F2, KISS1, NTS, F3, IGF1R, RHOA, PLAT, EPO, HMGB1, EDNRA, SELP, PTEN, CTSB, APOB, LDLR, HRAS, PIK3CA, PIK3R1, VEGFA, KDR, INS, AGTR1, HIF1A, TBXA2R, EDNRB
3	8.691	82	352	CHEK1, TGFB2, CDK6, ADRA2C, RNASE2, RNASE3, GCG, ADRA2A, RHEB, ADRA2B, NPY, NF1, ITGAL, LPA, EPHA2, BPI, ERBB4, SYK, HSP90AB1, CTSG, ADRB1, GC, CTSK, CTSL, PRL, CALCA, GLA, FGG, RBP4, ZAP70, PON1, PPARA, NES, PIK3CG, VDR, LEPR, HP, CST3, F5, DRD1, FABP4, ARG1, HMGCR, MMP12, OLR1, DRD5, EIF4E, AMBP, POMC, CRH, HSPA1A, RAC2, ATIC, BACE1, FABP5, HSPA8, BTK, AFP, ARSA, IGFBP1, HPRT1, GM2A, ANG, HEXB, SERPINC1, MAP2K3, KLF4, IMPDH1, F7, TGFBR1, CALCR, AKR1B1, VIP, ADM, INSR, NTRK1, CASP7, TGM2, APOC3, APCS, ESR2, RAC1
4	7.639	62	233	MMP8, JAK3, ATM, SERPINF2, AGER, S100B, CDKN3, NR3C1, XIAP, SERPINA1, COG2, MEN1, ADRB3, SERPIND1, FGFR1, ADRB2, SDC2, AGTR2, APOH, NOS1, PTK2, PTH, PPBP, NOS2, LCK, TTR, CFD, SELL, GDNF, CSK, THBD, B2M, APAF1, DRD2, MBP, NQO1, DRD3, DRD4, GSK3B, ALDOA, CYBA, MAPK10, FBN1, CDK2, P2RY12, PTPN11, IRS1, PSAP, PTPN1, ABL1, TGFB3, APOA2, APOA1, F13A1, NFE2L2, GSR, TEK, BMP2, FGF1, APLNR, APLN, LPL
5	5.75	9	23	ITGB1BP1, STRIP2, HEG1, CCM2L, RAP2A, PDCD10, STK25, MOB4, STRN
6	4.857	8	17	EPHX2, GART, DHFR, ME2, SHMT1, TPI1, TYMS, WARS
7	4	4	6	PDE4D, NT5M, PDE4B, DCK
8	3.789	20	36	AQP2, NEDD4L, ARF1, MAP3K3, SCNN1G, CYP17A1, SLC12A1, WNK1, RAB5A, XDH, SORD, UCK2, HSD11B2, UMPS, ADK, TK1, KCNJ1, CYP11B1, KIF11, PNP
9	3.5	17	28	HSD11B1, IMPDH2, GMPR, GSTA3, NPPA, ADH1B, NPPC, CYP2D6, SLC6A2, PDE5A, APRT, EPHX1, ADH1C, PNMT, GSTO1, GMPR2, NR3C2
10	3.375	33	54	GSTA1, KLKB1, SULT2A1, PRKACA, ACE2, F12, DUT, SOD3, PAPSS1, CYP19A1, CHIT1, ACTA2, MAP2, ADAMTS13, CTSS, QPCT, BMP7, BRAF, LTA4H, GLRX, F10, AGTRAP, SGK1, CRYZ, CDA, GSTP1, FKBP1A, CALM1, PDPK1, MTHFR, F11, PLK1, AURKA
11	3.333	4	5	RXRA, RARA, NR1H3, PCK1
12	3.333	4	5	CORIN, NPR2, NME2, NPR1
13	3	3	3	GPX3, GSTM2, GSTM1
14	3	7	9	HABP2, CYP2C8, CYP3A5, PAH, CYP2C9, NR1H4, PROZ
15	3	3	3	HBA2, CA2, HBA1
16	3	15	21	ALAD, AHCY, RAN, CLPP, ALDH2, YARS, FECH, PYGL, AGXT, DHODH, ACADM, PKLR, GPI, GLO1, HADH
17	3	3	3	RARB, RXRB, THRB
18	3	3	3	GNPDA1, GNPDA2, GALE
19	3	3	3	AKR1C1, CBR1, AKR1C2
20	3	3	3	AMY1B, AMY1C, HTN3

Cluster 1 is related to inflammation, smooth muscle proliferation, platelet activation, blood pressure regulation, angiogenesis, hypoxia, inflammatory response of leukocytes, vascular endothelial cells, vasodilation, vascular remodeling, and neuronal apoptosis. Cluster 2 is associated with the positive regulation of cytosolic calcium ion concentration, hypoxia, angiogenesis, vasoconstriction, blood pressure regulation, leukocyte activation and migration, platelet activation, axonal injury, synaptic transmission, neuronal apoptosis, and iron metabolism. Cluster 3 is mainly involved in platelet activation, blood pressure regulation, and glucose homeostasis. Cluster 4 is associated with platelet degranulation, hypoxia, blood pressure regulation, synaptic transmission, vasodilation, redox, and endothelial apoptosis. Cluster 5 is involved in angiogenesis. Cluster 10 is associated with coagulation, fibrinolysis, blood pressure regulation, redox, and hypoxia. Clusters 6, 7, 8, and 9 failed to return any ICH-related biological processes. The details were shown in Supplementary Table S4.

**Figure 5:**
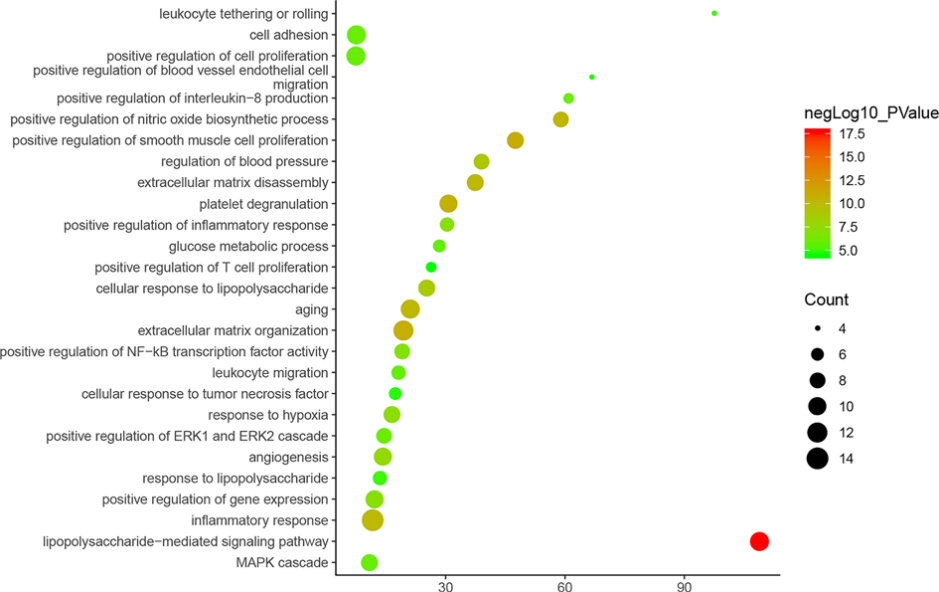
Bubble chart of biological processes of cluster 1 (*X*-axis stands for fold enrichment)

As the biological process of cluster 1 is more typical, it is shown as an example in Figure 5.

#### Signaling pathways of DH-ICH PPI network

**Figure 6:**
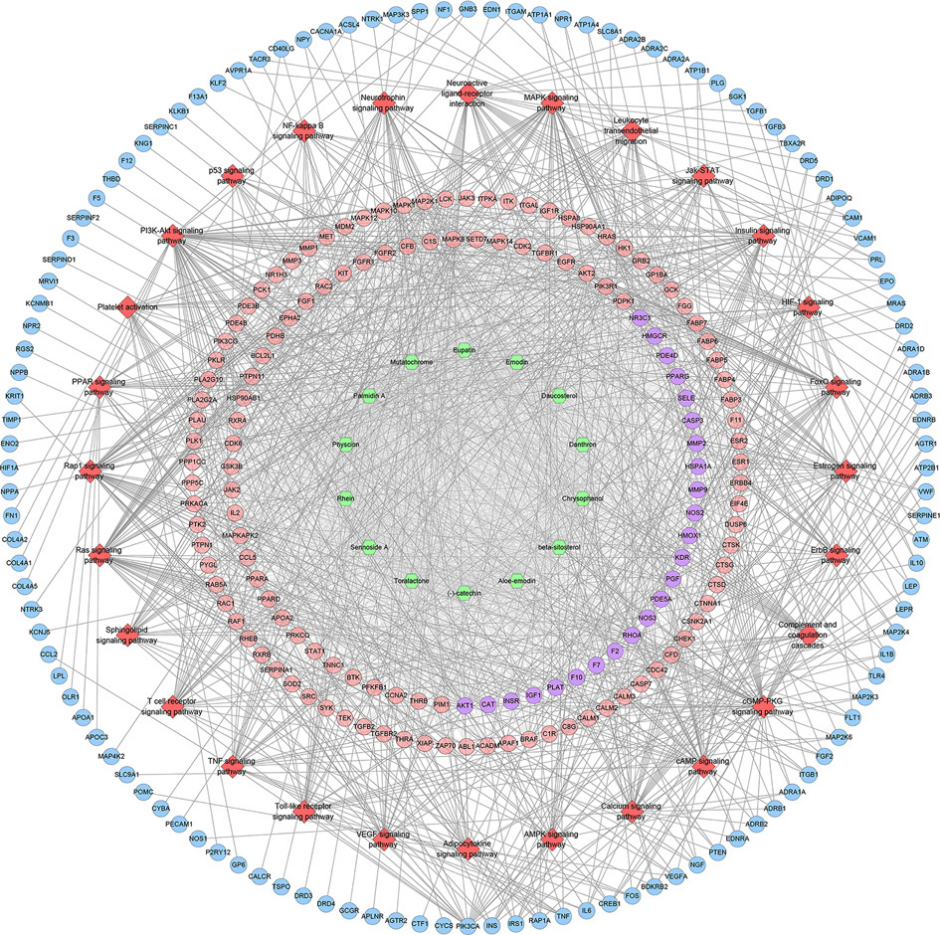
Signaling pathways of DH-ICH PPI network Green hexagons stand for herbs. Red diamonds stand for signaling pathways. Pink, blue, and purple circles stand for DH targets, ICH genes, and DH-ICH targets, respectively.

**Figure 7:**
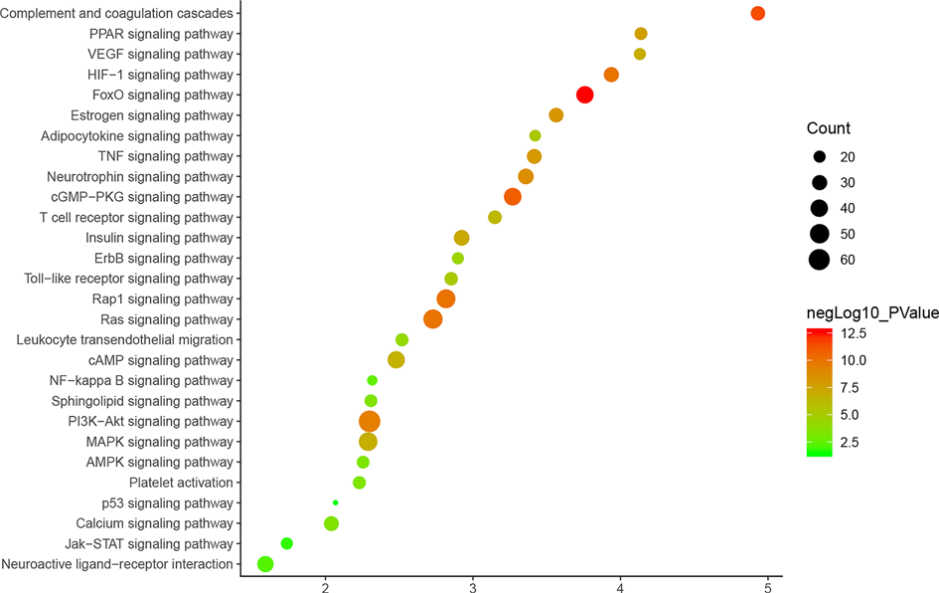
Bubble chart of signaling pathways (*X*-axis stands for fold enrichment)

The potential targets and ICH genes in DH-ICH PPI network were input into DAVID for signaling pathway enrichment analysis, and 28 ICH-related signaling pathways were obtained (Figure 6). The *P*-value, fold enrichment, and count of those signaling pathways were shown in Figure 7. Meanwhile, the number of targets regulated by different compounds is different (for the detail information, see Supplementary Table S5). The compound nodes were sorted in descending order according to their degree, as follows: Sennoside A (115 edges), Palmidin A (112 edges), Emodin (111 edges), Physcion (109 edges), Rhein (109 edges), Toralactone (109 edges), Mutatochrome (109 edges), Eupatin (107 edges), Aloe-emodin (105 edges), (-)-catechin (103 edges), β-sitosterol (97 edges), Chrysophanol (97 edges), Daucosterol (97 edges), and Danthron (63 edges).

#### Reactome pathways of DH-ICH PPI network

**Figure 8:**
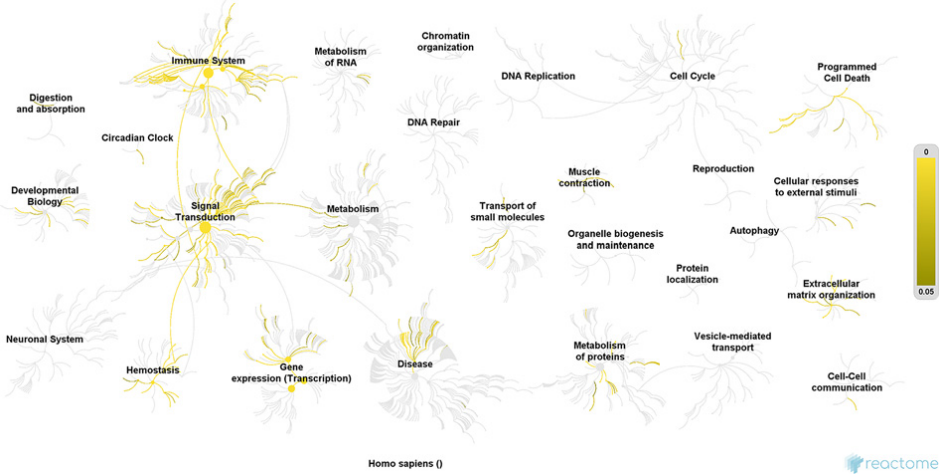
Reactome pathways of targets and genes (yellow from dark to light, indicating *P* value from 0.05 to 0)

**Figure 9:**
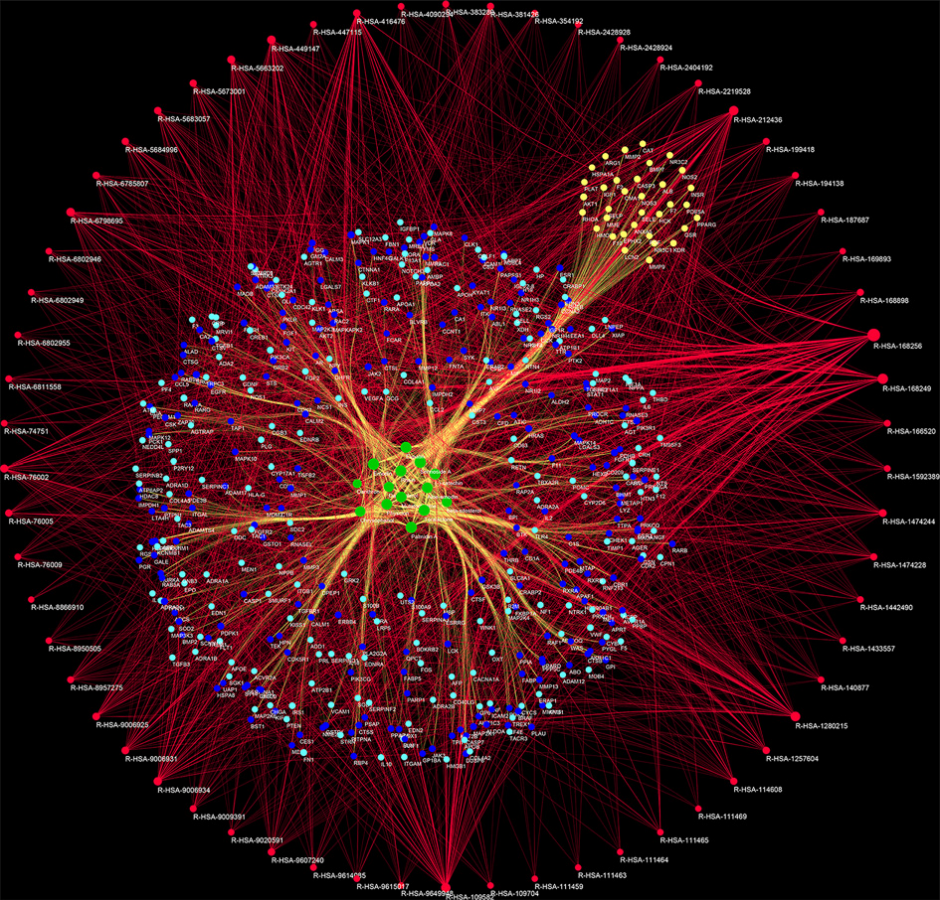
Reactome Pathways of DH-ICH PPI network Red circles stand for reactome pathway. Dark blue circles stand for DH targets. Light blue circles stand for ICH genes. Yellow circles stand for DH-ICH targets. Green circles stand for *Radix Rhei Et Rhizome* compounds. The larger the node size, the higher the degree of the node. The thicker the line, the greater the Edge Betweenness of the node.

The potential targets and ICH genes in DH-ICH PPI network were input into Reactome for reactome pathway enrichment analysis, and a lot of reactome pathways were returned (Figure 8). After screening, forty-six (46) ICH-related reactome pathways were returned (Figure 9).

**Figure 10:**
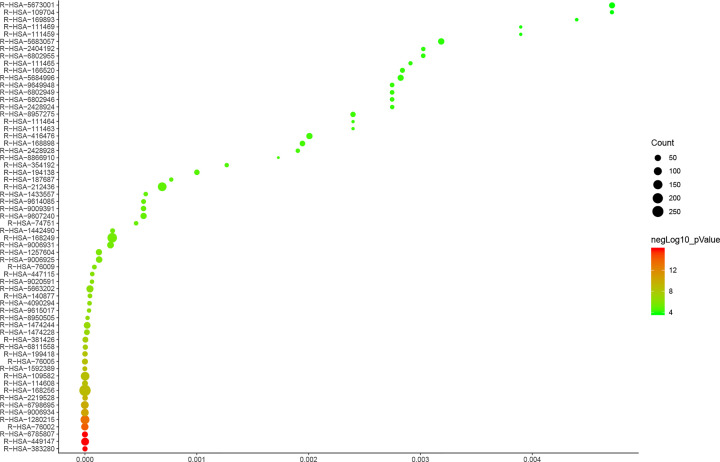
Bubble chart of reactome pathways (*X*-axis stands for FDR)

These reactome pathways were sorted in ascending order of *P* value and descending order of Count. The top 10 pathways are: (R-HSA-449147) Signaling by Interleukins, (R-HSA-6785807) Interleukin-4 and Interleukin-13 signaling, (R-HSA-383280) Nuclear Receptor transcription pathway, (R-HSA-76002) Platelet activation, signaling and aggregation, (R-HSA-1280215) Cytokine Signaling in Immune system, (R-HSA-9006934) Signaling by Receptor Tyrosine Kinases, (R-HSA-6798695) Neutrophil degranulation, (R-HSA-2219528) PI3K/AKT Signaling in Cancer, (R-HSA-168256) Immune System, (R-HSA-1592389) Activation of Matrix Metalloproteinases. The *P*-value, FDR, and count of those signaling pathways were shown in Figure 10 (Supplementary Table S6). Meanwhile, the number of targets regulated by different compounds is different. The compound nodes were sorted in descending order according to their degree, as follows: Palmidin A (205 edges), Toralactone (197 edges), Sennoside A (197 edges), Rhein (194 edges), Emodin (194 edges), Eupatin (193 edges), Physcion (188 edges), (-)-catechin (188 edges), Aloe-emodin (184 edges), Mutatochrome (182 edges), Chrysophanol (167 edges), β-sitosterol (162 edges), Daucosterol (158 edges), Danthron (108 edges).

The present study analyzed the network related to ICH intervention by *Radix Rhei Et Rhizome* and found that the core targets that it can regulate are: ZAP70, TYMS, TTR, TGM3, SYK, SULT2A1, SRC, RXRA, REN, PTPN1, PRKACA, PLA2G2A, PIM1, PDPK1, PDE5A, PDE4D, PDE4B, NR3C1, NR1H4, NQO1, NOS3, et al. The results of the biological module (namely, cluster) analysis showed that *Radix Rhei Et Rhizome* can interfere with ICH-related biological processes such as: is related to inflammation, smooth muscle proliferation, platelet activation, blood pressure regulation, angiogenesis, hypoxia, inflammatory response of leukocytes, vascular endothelial cells, vasodilation, vascular remodeling, neuronal apoptosis, and so on. The signaling pathway enrichment analysis also shows that *Radix Rhei Et Rhizome* can regulate many ICH-related signaling pathways, such as: FoxO signaling pathway, Complement and coagulation cascades, cGMP-PKG signaling pathway, Rap1 signaling pathway, HIF-1 signaling pathway, Ras signaling pathway, PI3K-Akt signaling pathway, Neurotrophin signaling pathway, Estrogen signaling pathway, TNF signaling pathway. The results of reactom pathway enrichment analysis show that the reactome pathway regulated by *Radix Rhei Et Rhizome* is related to interleukin signaling and its signaling pathway, platelet activation, neutrophil degranulation, blood coagulation and fibrinolysis, FOXO-mediated oxidative stress, metabolism and transcription of neuronal genes, the VEGFA–VEGFR2 pathway, dopamine receptor, MyD88/Toll signaling pathway; PI3K/AKT signaling pathway and so on. Experimental studies also showed that Radix Rhei Et Rhizome can regulate oxidative stress, programmed cell death (apoptosis, autophagy, etc.), and neurotrophic biological modules [36–40].

Recent research also confirmed some of the findings of the present study. In terms of inhibiting inflammation, emodin can promote microglia apoptosis by inhibiting the levels of IL-1 β, TNF-α, and increasing caspase 3 and 7 [41]. In terms of oxidative stress, emodin can increase the production of reactive oxygen species (ROS) and induce apoptosis of inflammatory microglia via Akt/FOXO3 [36]. Rhein can reduce oxidative stress by inhibiting the extracellular regulated kinase (ERK)/matrix metalloproteinase-9 (MMP-9) pathway [42]. Rhein also improved the superoxide dismutase (SOD) and catalase (CAT) activities, increased glutathione (GSH) levels and the glutathione/glutathione disulfide (GSSG) ratio, and reduced levels of malondialdehyde (MDA) and GSSG in rat with traumatic brain injury [43]. In terms of protecting the blood–brain barrier, *Radix Rhei Et Rhizome* or its active compound (emodin, rhein, chrysophanol) can attenuate the destruction of the blood–brain barrier by increasing the expression of zonal closure protein-1 in rats with ICH [44]. Rhubarb can also maintain the integrity of the blood–brain barrier and reduce the swelling of astrocyte foot processes by inhibiting the expression of the AQP-4 gene [45]. *Radix Rhei Et Rhizome* can down-regulate MMP-9 and up-regulate ZO-1 by inhibiting the ERK signal pathway [46]. In terms of vasodilation, emodin attenuates the production of NO in mice after explosive-induced traumatic brain injury by inhibiting the expression and activity of inducible nitric oxide synthase (iNOS), thereby reducing brain damage and improving behavioral scores [47]. Interestingly, some compounds of *Radix Rhei Et Rhizome* have similar effects, and the target sets between those compounds also overlap, which may be related to the main active compound being anthraquinones. For example, chrysophanol and rhein have almost the same molecular structure, except that one methyl group of chrysophanol is replaced by a carboxyl group; they can reduce the expression of caspase-3 and increase the activity of SOD in cerebral ischemic stroke models [48,49]. Same as rhein and chrysophanol, emodin removes one hydroxyl group and becomes chrysophanol; in cerebral ischemic stroke, they can reduce TNF-α, IL-1, and other inflammatory factors [50–52]. The present study also revealed the neuroprotective activity of anthraquinones from the perspective of chemoinformatics, and theoretically analyzed the mechanism of monomer compound interactions for treating ICH. In the future, further research is needed to confirm the mechanism of anthraquinone interactions in *Radix Rhei Et Rhizome* to treat ICH, and to find the best combination of anthraquinone monomers.

The mechanism of *Radix Rhei Et Rhizome*’s intervention in ICH has been predicted above using network pharmacology strategies. In order to verify the above results and further explore the molecular mechanism of *Radix Rhei Et Rhizome* treatment of ICH, the previous proteomics data will be analyzed in depth below. The proteomics data come from reference [53].

### Proteomics proteins’ PPI network analysis

#### Proteomics proteins’ PPI network construction

**Figure 11:**
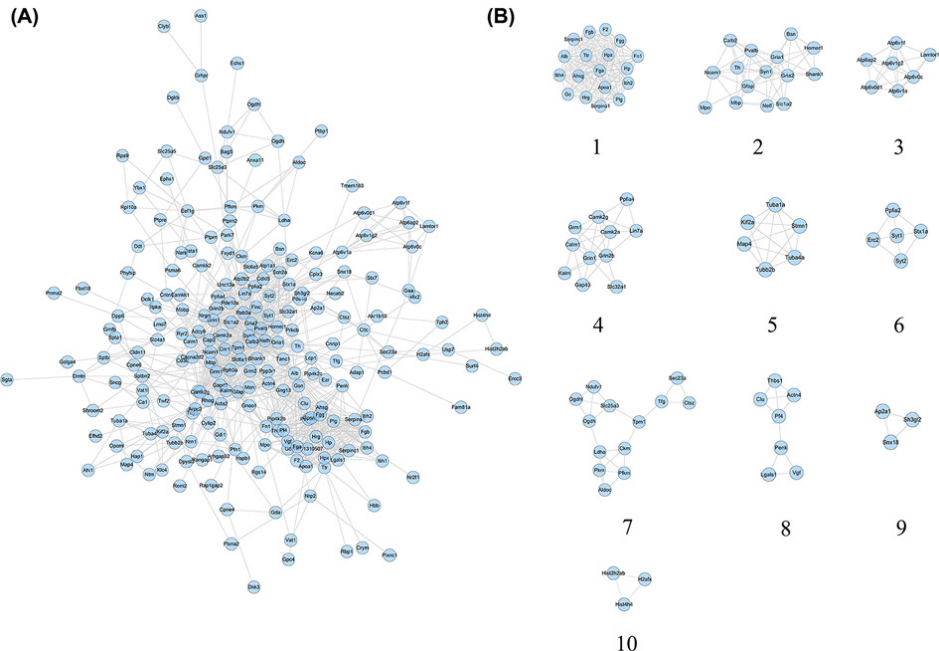
Proteomics proteins’ PPI Network Analysis (**A**) Proteomics proteins’ PPI Network. (**B**) Clusters of Proteomics proteins’ PPI Network.

The proteomics protein of reference [48] were shown in Supplementary Table S2. This proteomics proteins’ PPI network consists of 224 proteomics protein nodes and 976 edges (Figure 11A). The top 14 proteins are: Alb (51 edges), Syn1 (37 edges), Gria1 (37 edges), Syt1 (30 edges), Grin1 (30 edges), Pvalb (29 edges), Fn1 (29 edges), Calm1 (29 edges), Camk2a (28 edges), Grin2b (26 edges), Gria2 (26 edges), Gfap (26 edges), Fgg (26 edges), Apoa1 (26 edges) (Figure 9). This network was analyzed by MCODE, and 10 clusters returned (Figure 11B).

#### Enrichment analysis of proteomics proteins’ PPI network

**Figure 12:**
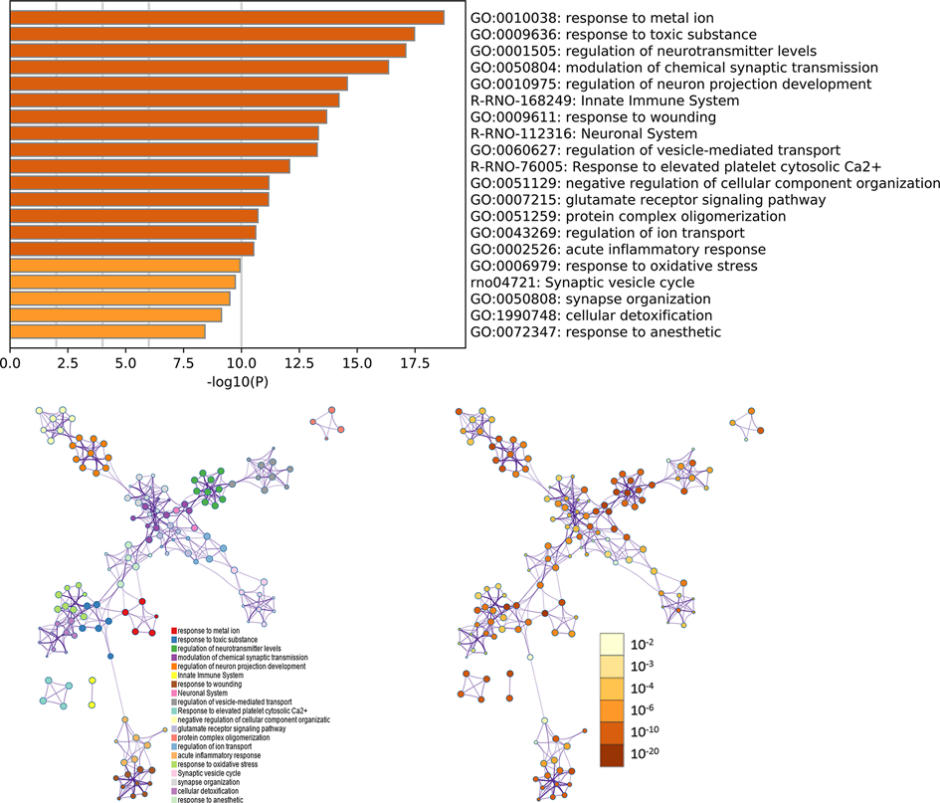
The main biological processes, signaling pathways, and reactome pathways

All proteomics proteins were input into DAVID and metascape (http://metascape.org/gp/index.html#/main/step1) for enrichment analysis. The biological processes, signaling pathways, and reactome pathways were shown in Supplementary Table S7 and Figure 12. The GO enrichment analysis showed that *Radix Rhei Et Rhizome* can interfere with multiple disease modules of ICH. For example: (1) Nerve cell transmission information module: chemical synaptic transmission, regulation of neurotransmitter levels, modulation of chemical synaptic transmission, regulation of trans-synaptic signals, activation of NMDA receptors and post-synaptic events, synaptic vesicle circulation, and so on. (2) Neuron module: regulation of neuronal projection projection development, regulation of neuronal differentiation, positive regulation of neuronal projection development, and so on. (3) Calcium ion module: calcium ion response, response to elevated platelet cytosolic Ca^2+^, and so on. (4) Excitatory and inhibitory amino acid modules: glutamate synapse, release NMDA receptor, glutamate binding and activation, glutamate receptor signaling pathway, and so on. (5) Coagulation module: platelet activation, coagulation, and so on. (6) Fibrinolytic module: fibrinolytic. The results of signaling pathway enrichment analysis showed that *Radix Rhei Et Rhizome* can regulate (rno04721) Synaptic vesicle circulation, (rno04724) glutamate synapse, (rno046100) complement and coagulation cascade, and so on. The results of reactome enrichment analysis showed that *Radix Rhei Et Rhizome* can regulate (R-RNO-112316) Neuronal System, (R-RNO-112315) Transmission across Chemical Synapses, (R-RNO-76005) Response to elevated platelet cytosolic Ca^2+^, (R-RNO-114608) Platelet degranulation, (R-RNO-109582) Hemostasis, (R-RNO-76002) Platelet activation, signaling and aggregation, (R-RNO-442755) Activation of NMDA receptors and postsynaptic events, (R-RNO-382551) Transport of small molecules, (R-RNO-111997) CaM pathway, (R-RNO-111933) Calmodulin induced events, (R-RNO-1489509) DAG and IP3 signaling, (R-RNO-422475) Axon guidance, (R-RNO-442729) CREB1 phosphorylation through the activation of CaMKII/CaMKK/CaMKIV cascasde, (R-RNO-9619229) Activation of RAC1 downstream of NMDARs, (R-RNO-111932) CaMK IV-mediated phosphorylation of CREB, (R-RNO-112314) Neurotransmitter receptors and postsynaptic signal transmission, (R-RNO-373760) L1CAM interactions, (R-RNO-438066) Unblocking of NMDA receptors, glutamate binding and activation, (R-RNO-112310) Neurotransmitter release cycle, (R-RNO-3858494) β-catenin independent WNT signaling, (R-RNO-5578775) Ion homeostasis. Experimental studies have also shown that *Radix Rhei Et Rhizome* can regulate nerve-related modules, oxidative stress modules, and extracellular matrix-related modules [54–57].

Compared with the predicted results, it can be found that the two have common biological processes with high enrichment, such as coagulation module, fibrinolytic module, neuronal synaptic plasticity, inflammatory factors and inflammatory cells, calcium ion module, oxidative stress, and iron metabolism. The common signaling pathways are: complement and coagulation cascades, Rap1 signaling pathway, estrogen signaling pathway, cAMP signaling pathway, and calcium signaling pathway. The common reactome pathways are: platelet activation, signaling and aggregation, signaling by receptor tyrosine kinases, neutrophil degranulation, hemostasis, platelet degranulation, response to elevated platelet cytosolic Ca^2+^, formation of fibrin clot (clotting cascade), intracellular signaling by second messengers, innate immune system, FLT3 Signaling, signaling by VEGF, post-translational protein phosphorylation, MAPK1/MAPK3 signaling, MAPK family signaling cascades, and RAF/MAP kinase cascade. In addition, proteomics enrichment analysis also revealed more biological processes, signaling pathways, and reactome pathways, see Supplementary Table S7.

## Conclusion

*Radix Rhei Et Rhizome* may intervene in biological process (such as inflammation, smooth muscle proliferation, platelet activation, blood pressure regulation, angiogenesis, hypoxia, and inflammatory response of leukocytes), signaling pathway (such as FoxO signaling pathway, complement and coagulation cascades, cGMP-PKG signaling pathway, and Rap1 signaling pathway), and reactome pathway (such as signaling by interleukins, interleukin-4 and interleukin-13 signaling, nuclear receptor transcription pathway, and platelet activation), so as to achieve the effect of treating ICH-related injuries.

## Supplementary Material

Supplementary Tables S1-S7

## Data Availability

The data that support the findings of this study are openly available in Supplementary Materials.

## Abbreviations

CAT: catalase
ERK: extracellular regulated kinase
GO: Gene Ontology
GSH: glutathione
GSSG: glutathione/glutathione disulfide
ICH: intracerebral hemorrhage
MDA: malondialdehyde
MMP-9: metalloproteinase-9
ROS: reactive oxygen species
SOD: superoxide dismutase
